# Exploring patient experiences with a telehealth approach for the PRO-ACTIVE trial intervention in head and neck cancer patients

**DOI:** 10.1186/s12913-022-08554-6

**Published:** 2022-09-30

**Authors:** M. M. Khan, B. Manduchi, V. Rodriguez, M. I. Fitch, C. E. A. Barbon, H. McMillan, K. A. Hutcheson, R. Martino

**Affiliations:** 1 Department of Speech-Language Pathology, University of Toronto, 160-500 University Avenue, Toronto, ON M5G 1V7 Canada; 2 Krembil Research Institute, University Health Network, Toronto, ON Canada; 3 Rehabilitation Sciences Institute, University of Toronto, Toronto, ON Canada; 4 Bloomberg Faculty of Nursing, University of Toronto, Toronto, ON Canada; 5 Department of Head and Neck Surgery, The University of Texas MD Anderson Cancer Center, 7007 Bertner Ave., Houston, TX 77030 USA; 6 Department of Radiation Oncology, The University of Texas, M.D. Anderson Cancer Center, Houston, TX USA; 7 Department of Otolaryngology- Head and Neck Surgery, University of Toronto, Toronto, ON Canada

**Keywords:** Telehealth, Dysphagia, Head and neck cancer, Patient experience, Rehabilitation

## Abstract

**Introduction:**

Following the COVID-19 directive to cease non-essential services, a rapid shift was made in the delivery of Speech Language Pathology (SLP) dysphagia management in the 3-arm, randomized PRO-ACTIVE trial. To inform future programs, this study explored patients’ experiences with telehealth when the planned in-person SLP intervention was moved to a telehealth modality.

**Methods:**

A theory-guided qualitative descriptive approach was used. Willing participants who had received at least one telehealth swallowing therapy session participated in a one-time semi-structured interview. Interview transcripts were subjected to a standard qualitative content/theme analysis. Researchers reviewed all transcripts and used a multi-step analysis process to build a coding framework through consensus discussion. Summaries and key messages were generated for each code.

**Results:**

Eleven participants recounted their telehealth experiences and reported feeling satisfied, comfortable and confident with the session(s). They identified that previous experience with teleconferencing, access to optimal technical equipment, clinician skill, and caregiver assistance facilitated their telehealth participation. Participants highlighted that telehealth was beneficial as it reduced commuting time, COVID-19 exposure and fatigue from travel; and also allowed caregiver participation particularly during COVID. In comparing their in-person SLP sessions to telehealth sessions, limitations were also identified, including: lack of previous experience with and/or poor access to technology, and less opportunity for personalization. Participants indicated that use of phone alone was less preferred than an audio/video platform.

**Discussion:**

Patients reported that overall, telehealth sessions did not compromise their learning experience when compared to in-person sessions. Patients benefited from use of telehealth in several ways despite some limitations of the use of technology. Patient feedback about telehealth provides an important perspective that may be critical to inform best practices for care delivery.

**Supplementary Information:**

The online version contains supplementary material available at 10.1186/s12913-022-08554-6.

## Introduction

On March 11, 2020, the World Health Organization declared the novel coronavirus disease (COVID-19) as a pandemic. This prompted healthcare professionals to reduce and/or cease non-essential medical services, wherever possible, across the world. Head and neck cancer service, in particular, was challenged with the difficult task of balancing between COVID-19 infection risk mitigation and risk of cancer disease progression [1, 2]. Supportive services including dysphagia management for head and neck cancer patients were largely affected [3–5] since both swallowing evaluation and treatment sessions involve close physical proximity between patients and their clinicians, including several aerosol-generating procedures [4, 6]. Accordingly, the conduct of ongoing clinical trials was also severely impacted [7], including the PRO-ACTIVE trial (Prophylactic Swallow Intervention for Patients Receiving Radiotherapy for Head and Neck Cancer) [8, 9].

PRO-ACTIVE is a large multi-site, 3-arm, pragmatic randomized controlled trial, evaluating the effectiveness of prophylactic swallowing therapy for head and neck cancer patients undergoing radiation therapy [9]. In the PRO-ACTIVE trial, patients are randomized to receive one of three behavioural swallowing interventions: a low intensity face-to-face swallowing therapy with an SLP that starts before radiation therapy, continues bi-weekly during radiotherapy and includes a clinical swallowing assessment, education on symptom management, and a structured program (EAT-RT) to facilitate safe but challenging oral intake [10]; a high intensity swallowing therapy that is similar to the EAT-RT program but with the addition of swallowing exercises conducted in between meals; and, a control group who is offered the high intensity therapy late during radiotherapy but only to patients who develop swallowing issues while receiving radiation therapy. The PRO-ACTIVE trial launched in late 2018 with the aim to recruit 952 patients in five-years across seven institutions in Canada and the United States. When COVID-19 was declared a pandemic in March 2020, the trial had been open for 16 months and had enrolled 29.6% (*n* = 282) of the target sample.

At the start of the pandemic directive, there were 35 participants in the active intervention phase of the trial, 203 being followed post radiation therapy and 44 who had either completed or withdrawn from all study activity. At all study sites, health care and research services shifted to a telehealth approach wherever possible to reduce person-to-person contact. For the PRO-ACTIVE trial, the COVID-19 directive triggered the need for a rapid adaptation in how swallowing intervention was delivered to trial participants. Of special concern was how to continue to offer swallowing therapy to those who were actively receiving it during their radiation therapy. Also, the shift to telehealth needed to be rapid in order to meet the rigid research protocol timelines.

To meet this urgent dynamic shift, the authors promptly designed a telehealth approach for use within the PRO-ACTIVE trial based on literature reviewed concerning dysphagia telehealth and consultation with selected SLPs within the research project [11]. Although there is growing literature on telehealth practice across patient populations [12], there was at that time limited research evaluating telehealth for dysphagia management using an existing platform within a patient’s home environment. Despite this, there was evidence to endorse the feasibility of using a telehealth approach for dysphagia management in head and neck cancer [13] including studies that provide valid and reliable outcomes for dysphagia management comparable to those obtained in an in-person SLP session [14, 15]. A telehealth approach may provide an alternate model to support patients in areas with constraints for intensive in-person clinician-directed therapy [16]. It also has the potential to improve patient access to cancer care [17] and clinical trials participation [18].

Within the broader literature on telehealth, studies have reported on patient experiences with telehealth [19, 20] and attempted to isolate the facilitators and barriers to success in using the technology to deliver health care services from a remote setting [21–23]. Benefits to telehealth include the potential of technology to overcome geographic and cost issues [24], yet challenges may exist for patients related to accessing, adapting and/or accepting these technical factors [25]. In summary, the use of telehealth approaches is complex with many components to consider, including the need to understand more about the patient experience and engagement with the virtual platform.

In light of the need to rapidly deploy telehealth for our swallowing intervention in the PRO-ACTIVE trial, the authors based our current implementation on what was known from only the clinician perspective [11] and local institutions’ infrastructure. However, to ensure that implementation of the telehealth platform is successful in the future, it is important to learn what worked and did not work from the perspective of head and neck cancer patients themselves. Recipients of a service may in fact have different perspectives than those providing the service and their perspective can contribute to the success or failure of interventions [26, 27]. Such information would be essential to facilitate our ability to make course corrections in not only providing a behavioural swallowing intervention in the PRO-ACTIVE trial but also provide patient-centered high quality integrated supportive care and rehabilitation in the head and neck cancer population [28]. Therefore, the objective of this study was to understand patient experiences from their perspective regarding telehealth interaction for swallowing therapy during radiation therapy as part of the PRO-ACTIVE trial. Patient feedback about telehealth will provide an important perspective critical to inform best practices for SLP care in head and neck cancer and inform future telehealth programs for health care delivery.

## Methods

A theory-guided qualitative descriptive approach was used, applying content analysis of individual semi-structured interview transcript data [26, 27, 29]. This involved identification of meaningful phrases and subsequently grouping them into common themes [30]. The aim of this process was to understand patient experiences from their perspective of the telehealth interaction implemented rapidly in response to COVID-19 restrictions within the PRO-ACTIVE trial. Individual interviews were conducted with participants following the completion of their swallowing intervention.

### Participants

To gain consent for this study, the interviewers used telephone or face-to-face interaction to approach all PRO-ACTIVE study participants who had received at least one telehealth swallowing therapy session between March and October 2020, from two PRO-ACTIVE study sites: the Princess Margaret Cancer Centre, part of the University Health Network (PM/UHN) and the MD Anderson Cancer Center (MDACC). PM/UHN is located in Toronto, Ontario and is the largest comprehensive academic cancer treatment facility in Canada. MDACC is located in Houston, Texas, USA, and is a world leading center devoted exclusively to cancer care, research and education. Full details on the eligibility criteria for the PRO-ACTIVE parent trial are provided elsewhere [8, 9]; however in brief, these were adult patients with head and neck cancer who were planned to receive bilateral radiation therapy at or above 60 gy and who had a functional swallow before starting their radiotherapy. Given that PRO-ACTIVE is a pragmatic trial, we did not exclude patients based on tumour stage or type, or any additional cancer treatment. Likewise, we broadly defined a telehealth session as any remote, real time communication that was not in person, including audio, video and/or a combination of these platforms, as dictated by local institutional infrastructure and patient’s access.

### Data collection

Participants who consented were invited to individual, semi-structured interviews led by one of four trained facilitators, authors MMK, BM, CEAB and HMcM. MF, as the senior qualitative expert, provided training to all facilitators. Three facilitators were SLP clinician researchers with either a Masters or doctoral degree. One facilitator was a research coordinator with Masters training. Of the remaining authors, two (RM and KAH) were SLP clinician researchers and one (MF) was a nurse researcher with doctoral degrees. VR was an SLP clinician researcher with Masters training. All eight authors were females affiliated with academic medical facilities.

To create a safe environment for the participants, all interviews were conducted by a researcher not directly involved with the delivery of PRO-ACTIVE swallowing therapy sessions for that individual. The interview script included open-ended questions focused on participant’s experience with telehealth specifically for the PRO-ACTIVE swallowing therapy provided by a clinical SLP. The key messages targeted included: their expectations, acceptability, facilitators, barriers, benefits and drawbacks regarding the use of telehealth. The interview guide developed for this study is provided as Additional File 1. All interviews were conducted and recorded using institutionally approved audio/video (A/V) platforms, such as MS Teams or WebEx, at a time convenient to the patient and scheduled for thirty minutes. Ethical approval was obtained from the Research Ethics Board (REB) from each of the participating sites.

### Data analysis and reduction

All interviews were transcribed verbatim, using an automated transcription software (Otter.ai - Otter Voice Meeting Notes) then cross checked manually for accuracy, and de-identified. The final transcripts were analyzed using a standard content/theme analysis [31]. The team of researchers with collective expertise in the clinical care of head and neck cancer patients, SLP practice and/or qualitative analysis, planned the multi-step analysis process. In step one, 20% of the transcripts from the PM/UHN site were randomly selected for independent review by two raters (MK, MF) with the aim to generate coding categories. Step two, these researchers met to discuss their observations and reach consensus regarding the content identified and how the content ‘fit’ to build a coding framework. Step three, the remaining researchers (BM, VF, RM) independently reviewed the same transcripts and applied the coding framework derived in step two. Step four, all researchers met to finalize the coding framework through consensus discussion. Step five, the final coding framework was applied to all interviews at PM/UHN with each transcript independently reviewed by two of the trained raters. Step six, the paired raters met to discuss their coded transcripts, reconcile any discrepancies through consensus, and identify new codes that may not have been previously identified. Step seven, all raters met to review and discuss the data, with the aim to reach consensus on the final analysis and major content categories. Step eight, each rater was assigned one or more major category and they independently generated a brief summary and key messages for each assigned category by reviewing all transcripts and borrowing from the participants’ voice. Step nine, to ensure accountability, all raters met to discuss and agree on key messages for each coding category.

The same process was repeated with the interview transcripts from the MDACC site when they became available. Interviewers from MDACC conducted the interviews which were locally transcribed, cleaned and de-identified before being exported for analysis by the raters at PM/UHN. Steps one to eight were enacted as per above and step nine included the MDACC facilitators to discuss and agree on key messages. Part of the discussion focused on identifying any observed differences in meaningful content between the PM/UHN transcripts and the MDACC transcripts.

## Results

### Participants and session characteristics

Sixteen eligible patients were approached from the two lead sites, of which eleven (age: 58.5 + 8.7, 63.6% male) consented and were interviewed: seven from PM/UHN and four from MDACC. Across all participants who had received SLP swallowing therapy sessions via telehealth: six received low intensity swallowing intervention (EAT-RT) and five high intensity swallowing intervention (EAT + Exercise). The number of telehealth SLP therapy sessions completed by each participant ranged between 1 and 4 with majority (eight out of eleven) completing 1–2 sessions. Eight of the eleven patients had received at least one in-person SLP therapy session prior to transitioning to telehealth. Also, eight participants received telehealth sessions using an A/V platform, and the remaining three participants received telehealth sessions using phone only or a combination of phone and A/V platform. Participants were interviewed between 3 to 9 months following their last telehealth SLP therapy session. On average, the interviews lasted 20 minutes. These details along with participant demographics are detailed in Table 1.

**Table 1:** Participant demographics, telehealth sessions and interview details

	Participants (***n*** = 11)
**Demographics**	
Age, mean years +SD	58.5 + 8.7
Male, n (%)	7 (63.6)
Canada/USA, n (%)	7 (63.6)/4 (36.4)
**Telehealth SLP sessions per participant**	
Telehealth via A/V, n (%)	8 (73%)
Telehealth via phone + A/V, n (%)	2 (18%)
Telehealth via phone only, n (%)	1 (9%)
**Interview sessions per participant**	
Time between last telehealth session and interview, mean months +SD	4.9 + 2.5
Length of interview, mean minutes +SD	19.6 + 7.0

### Participant perspective regarding telehealth

Participants were able to share descriptions of their telehealth SLP swallowing therapy sessions and recount their expectations and evaluations of those sessions. They identified factors that facilitated their telehealth sessions, and concerns regarding the limitations and barriers to telehealth comparing, if appropriate, their in-person and telehealth experiences. In addition, participants identified benefits to telehealth particularly in light of the COVID-19 pandemic. Some offered suggestions on how to improve the overall SLP-led telehealth therapy session with the potential for future implementation. Several common key messages, emerged during the analysis. The main points for each key message are provided below. Table 2 provides an overview of topic areas including key messages summarized by researchers from participant viewpoint for each content category.

**Table 2:** Overview of topic areas, code categories and key messages

Topic areas	Code category(s)	Definition	Key message (themes) summarized from participant viewpoint^a^
What were the telehealth SLP sessions like?	Description of the telehealth session	Factual account of what happened during the session	The SLP watched my swallowing, then demonstrated the exercises or discussed the food chart and answered any questions I had. Sessions did not require much preparation and it was more informational.
	Participant expectation of PRO-ACTIVE telehealth session	What participant thought might happen during the session before the first telehealth session occurred	Overall I thought the idea of using telehealth was great when I heard about it, but I wasn’t sure what to expect at my session.
	Participant evaluation of PRO-ACTIVE telehealth session	Participant’s summative evaluation of the session/satisfaction; experience of using telehealth as a mode of care delivery	I felt satisfied and comfortable after the sessions. They worked well, were easy to follow, and I received clear instruction and guidance.
	Participant outcomes from PRO-ACTIVE telehealth session	Participants’ expressions of confidence and accomplishment at end of session/learning	I learned what I needed to learn and felt confident doing it on my own afterwards.
What worked well for telehealth SLP therapy sessions?	Facilitating factors of telehealth	What helped with participating in the telehealth sessions i.e. things (factors) that made it easy?	I’ve used teleconferencing before, for work and for medical appointments, and am quite comfortable with the technology. It was a huge plus to have a caregiver present and have an engaging and responsive clinician lead the telehealth sessions.
What did not work for the telehealth SLP sessions?	Limitations in using telehealth	Perceived limitations in using telehealth as a mode of care delivery)	Possible limitations could be if I was not very technical as far as computers are concerned or did not have any previous experience receiving health care via telehealth.
	Barriers in using telehealth	Perceived barriers in using telehealth as a mode of care delivery – things that stopped or prevented the session from happening	Severe swallowing deficits, or having a lot of symptoms, or experiencing technical glitches, may possibly make telehealth more difficult for some patients.
	Drawbacks of using telehealth	Negative effects in using the telehealth approach	Teleconferencing does not allow for a close examination of the mouth and the throat and there is just a little something missing in these sessions that doesn’t allow for the nuances of an in-person session or an interpersonal connection.
	Participant concerns about telehealth	Participant worries or concerns about the telehealth approach	I was concerned about problems with my internet connection and whether the therapist could really see how I was swallowing or doing the exercises.
How does telehealth compare to in-person SLP sessions?	Comparing between in person and telehealth sessions	How did participants compare their in person with telehealth sessions	Telehealth sessions were comparable with no real difference to in-person sessions and just as effective. It did not compromise what I learned when compared to live sessions.
	Participant perception about having video option for PRO-ACTIVE telehealth session	Participants’ specific comments about having the video option	Video conferencing is better than telephone because it allows the therapist to show how to execute the exercises and to give patients feedback on those.
Benefits of using telehealth	Overall benefit of using telehealth	Benefits of using telehealth sessions as a mode of cancer care delivery	Convenience is a big factor. The telehealth sessions saved commuting time, and I wasn’t exhausted from it. It allowed having someone else participate and not wasting their time.
	Benefits of using telehealth during COVID	Benefits of receiving care using telehealth during COVID	During COVID, it was a super positive experience because every time I would go to the hospital for radiation or whatever, there was always a potential chance of meeting or interfacing with somebody who could contaminate me.
Suggestions to improve telehealth experience and optimize overall healthcare delivery	Suggestions to improve telehealth session	Specific suggestions or recommendations by the participants to improve telehealth sessions	Video works better than telephone. Also suggest using a video conferencing platform that is easy to use and allows recording of the sessions.
	Suggestions to improve overall PRO-ACTIVE SLP care delivery	Suggestions to improve overall PRO-ACTIVE SLP care delivery not related to telehealth or COVID	There isn’t anything to improve; the only suggestion is to ensure easy access to individual patient test result details and therapy instructions.
	Potential for future telehealth applications	Participant suggestions for implementing telehealth in areas of health care delivery in future	Use telehealth for team meetings with patients to make communication more efficient and improve care.

^a^ Key messages were summarized using the words from participants and reported using the first person to reflect what they would have said - they are illustrative examples of the summarized participants’ ideas and words

### Key message 1: what were the telehealth SLP therapy sessions like?

Participants recalled their telehealth sessions beginning with the SLP describing what would happen during the session. They had received a copy of the study materials describing the EAT-RT staircase +/− exercises (depending on trial arm) and would have them open during the session(s). Participants mentioned that the sessions did not require much preparation although some recalled being asked to bring various food textures (e.g., water, pudding, yogurt, and cookie) to use and demonstrate their swallowing status. Depending on the study arm and availability of an A/V platform, the SLP would demonstrate swallowing exercises and allow the patient to repeat them and provide feedback. On second and subsequent sessions, the SLP would inquire about participants’ progression with their swallowing, the food staircase and the exercises. Participants indicated they were able to mention if the material was unclear or confusing; they had the opportunity to ask questions and were able to receive explanations and feedback from the SLP. Only one participant described that their telehealth sessions did not evaluate their swallowing but were mainly informational, reinforcing what they had already learned in clinic. One participant had difficulty recalling the details of their telehealth session during their interview.

In terms of expectations, overall participants thought that the idea of using telehealth was great when they heard about it. Although some were not sure what to expect during the session, most indicated they had no concerns with switching to a telehealth platform. Participants evaluated their telehealth sessions as a very simple process where they felt supported and very comfortable. A couple of patients from both PM/UHN and MDACC reported that, initially, the session would start awkwardly, but as time progressed, they moved well. Overall, participants reported feeling satisfied and informed at the end of their telehealth SLP sessions. Depending on their trial randomization arm, they indicated they had learned how to eat the right foods and how to do the exercises correctly.

Illustrative quotes:

*We were going through every exercise while I was doing it. I would have water, different types of food; she would watch me eat and swallow and be able to give me pointers” [PT-06].*

*I was excited because it made my life a little bit easier [PT-11.].*

*I felt confident that I was doing the exercises as they were intended. And that I was, you know, informed on how to go ahead with how to eat certain foods as the food pyramid illustrated [PT-02].*

### Key message 2: what worked well for telehealth SLP therapy sessions?

Participants identified several factors that made it easy to participate in the telehealth sessions. Six of the eleven participants mentioned they were very comfortable with the use of technology and that they “know the system”. They indicated it was helpful to have previous experience using teleconferencing platforms at their respective professional careers although only two from this group had previously used a telehealth approach for healthcare delivery. Participants identified having good internet connection, and access to optimal equipment as potential facilitators for their telehealth session. Having a family member or caregiver support also helped facilitate the sessions; where one had the opportunity to have a partner provide assistance with technical support and also join the session to participate, take notes and provide feedback in real time.

Participants reported it was easy to participate in the session as there was minimal preparation required. They enjoyed talking to their clinicians who provided clear information with the right paperwork and instructions and helped troubleshoot technical issues as needed. A few participants reported it was helpful to have met the SLP at an in-person session beforehand and have a familiar face lead the telehealth session. Some also made note that the more telehealth sessions they did, the better they got at it. Finally, one participant attributed the ease of running the session via telehealth to their low symptomatology.

Illustrative quotes:

*I work in the technology industry, most of my conferences or calls or daily interactions with customers and or whatnot was through either a phone call video conferencing, so speaking to a medical professional wasn’t any big deal. [PT-08].*

*I could put him [the caregiver] on speaker and he could participate, because he would help me ask questions or sometimes, you know, he can see my face [if] I didn’t understand. So he would say, Are you sure you understand you ask the question again, and so we can help clarify. So it was, it was really good way to have your support there. So that was a huge plus. [PT-04].*

*I have one office downtown [and] I work from home. I already have my laptop, I bring [it] home all the time and I have good internet connection here. So I didn’t have to upgrade anything in terms of software or fiber or I don’t know my system. [PT-07].*

### Key message 3: what did not work for the telehealth SLP sessions?

Participants identified a few limitations in using telehealth. One limitation was their lack of previous experience using a teleconferencing platform for healthcare delivery and/or the general lack of experience with technology. Some reported being limited by inadequate access to optimal equipment. They wondered if the technology made it difficult for the SLP to really see how they were swallowing or completing the exercises. They saw one of the major challenges was that teleconferencing did not allow for a close examination of the mouth and throat. One participant indicated the type of software may be a limiting factor if it is not user-friendly. Another participant also pointed out that even though their experience with telehealth was good, it may be particularly challenging for patients who experience severe swallowing deficits, or those having a lot of treatment side effects. Lack of any caregiver support and the use of hearing aids were also perceived as potential limitations in using telehealth as a mode of care delivery.

Other drawbacks highlighted by participants regarding telehealth sessions included limited personalization, and the little opportunity to see patients and their clinician’s reactions including information conveyed through body language as you would find in any in-person session. Finally, participants indicated that telehealth is limiting when conducted just over the phone without video.

Illustrative quotes:

*I’m not very technical as far as computers are concerned [PT-05].*

*The one time we were having issues, it would freeze up. So everything was kind of chattering. So you would kind of have to wait. So that was a little frustrating [PT-06].*

*I really didn’t have a lot of negatives, the only thing is instead of just being telephone, instead of just being voice, it should also be video.[PT-04*].

### Key message 4: how does telehealth compare to in-person SLP sessions?

Of the eight participants who started with in-person sessions and then transitioned quickly to telehealth, their perceived comparison indicated that their telehealth sessions overall were comparable to in-person sessions. That is they stated that they were just as effective and did not compromise what they needed to learn. Only two of these participants indicated a preference for an in-person session and thought it would be much more beneficial than telehealth. These participants felt that seeing somebody in person makes a difference and close proximity allowed a joint brainstorming effect that is difficult to come across in a virtual session.

Even though most participants felt the effectiveness of SLP intervention was not compromised by the switch to telehealth, they valued the use of an A/V platform for their sessions and declared they preferred it over the phone channel. They thought an A/V channel allowed for a better connection with the therapist. It gave them the opportunity to show the therapist specific concerns related to the head and neck area, (e.g., swelling of the throat), benefit from a clear demonstration on how to execute the exercises, and to receive feedback on their performance.

Illustrative quotes:

*Effectiveness, I don’t, I don’t know if there’s a compromise in either way. I think, I like to think that they’re effectively getting the results that you’re looking for both ways. [PT-01].*

*[It] makes you feel better seeing them in person that way they can say, “Oh, it looks so good. And you’re doing well”. Yeah, they said the same thing when I talked to them [SLP] on the phone, but seeing somebody makes a difference, I guess. [PT-09].*

*As far as instruction about the exercises, who to keep working with, that was you know, on par with in clinic. I would say for both in clinic and the telehealth, you’re capturing it all. [PT-10]*

### Key message 5: benefits of using telehealth

Several benefits were identified in using telehealth for PRO-ACTIVE SLP sessions. In both US and Canada, participants mentioned how teleconferencing saved time and effort of driving every day from a distance and allowed them to continue participating in the sessions. Participants mentioned that teleconferencing relieved them from the stress, anxiety and physical hardship of getting to the hospital and it was a great benefit to be able to attend the session from the comfort of their home. For some participants, less fatigue implied less need to rely on caregivers. Multiple participants saw benefit in the opportunity to use the full time available for their SLP session by avoiding clinic waiting times. One participant mentioned the telehealth session allowed them to avoid embarrassing situations such as gagging, choking or needing to spit mucus in front of the clinician.

Canadian participants, in particular, reported a great relief in not having to come to the hospital for an SLP session during COVID. They supported the choice of shifting their SLP sessions from face to face to telehealth in light of the pandemic as it meant one less appointment and one less reason to be in a “*germy”* hospital [PT-05]. Shifting to telehealth guaranteed continuation of their SLP treatment without necessitating the exhaustive need for donning personal protective equipment (PPE) for every hospital visit as part of COVID-19 protocol. Lastly, due to COVID restriction, most hospitals had strict no visitor policies in place. Participants highly valued the opportunity provided by telehealth to allow a caregiver, often their partner, to listen in and participate in their SLP sessions.

Illustrative quotes:

*I think [the sessions] can be handled more efficiently actually, by the telehealth, like the commute, reducing the risk of getting COVID, having someone else participate and not wasting their time. All three big benefits of having it done through a virtual meeting [PT-07].*

*I was excited because it made my life a little bit easier, not having to drive to go to a visit. That we could do it from wherever, usually it was work, which made it easier. So all in all, I thought [it] was very beneficial for me to do that instead of going to in person [PT-11].*

*It was a huge help in keeping me participating without having to use a lot more energy or having to, you know, drive down there. You know, walk through all the washing hands and getting masks and you know what it was just like in the COVID environment, it was a huge relief. So for me, it was good. [PT-04].*

### Key message 6: suggestions to improve telehealth experience and optimize overall healthcare delivery

Overall, participants indicated being happy with their telehealth experience, and did not have many recommendations to change. They did indicate, if provided with a choice, they would opt for an A/V channel versus telephone only. Participants also suggested using a teleconferencing platform that is easy to use and allows recording as it would be a benefit to review the session at a later time. Other suggestions included ensuring easy access to individual patient test results along with detailed SLP therapy notes and recommendations; and the inclusion of a caregiver in SLP sessions.

In terms of future interventions, one participant suggested that telehealth mode of healthcare delivery could be useful for multidisciplinary team meetings where it allows several specialists to meet the patient at the same time, thereby improving efficiency in communication among healthcare team members resulting in better care. They also highlighted the potential to use telehealth to reach people living in remote areas that are often underserved by the health community.

Illustrative Quotes.

*Again, it depends on the study, but on this research that requires some exercise to be able to maintain your ability to swallow. I think video would have improved it. [PT-04].*

*Yeah, actually might be, or as the exercises go, you can replay the session and actually redo the session, have the session available to you to redo to review the exercises again, because when we think we remember, well, I’ve had some past experience of what we think we remember and you’re tested on it for a period of time, it’s not exactly the same. Being able to replay the session would be, I think, a benefit [PT-10].*

*I could see it being beneficial, having, you know, the different specialists in the same meeting so that they could compare notes and possibly bounce ideas off each other as well. So I guess just a sidebar note on how well teleconferencing can work in the health care sector [PT-02].*

## Discussion

This project aimed to understand patient experiences regarding telehealth interaction in a small subset of participants enrolled in the large PRO-ACTIVE trial. Several lessons were evident from the key messages that emerged from analysis of the participant interviews. Participants reported that telehealth sessions did not compromise with what they would have learned in an in-person session. Overall, participants felt satisfied, comfortable, and confident after their telehealth sessions. They identified that previous experience with teleconferencing, access to optimal technical equipment and caregiver assistance facilitated their telehealth participation. Telehealth was seen as beneficial as it saved commuting time and energy at a time when cancer patients are feeling physically vulnerable and weak. In comparing their in-person SLP sessions to telehealth SLP sessions, participants identified key limitations including a lack of previous experience, poor access to technology and limited personalization of a telehealth session. In particular, participants strongly indicated that use of telehealth is limiting when conducted just over the phone without video.

In recent years, several publications have emerged with positive findings on the feasibility and reliability of telehealth use in dysphagia management for head and neck cancer patients. Advantages reported include improvements in service efficiency [32], cost savings [13], access to care [33], and clinician and patient/caregiver satisfaction [13, 32], all of which aim to ultimately enhance overall patient care delivery [33]. However, in addition to investigating the feasibility and reliability of telehealth practice, it is also important to learn the perspective of head and neck cancer patients themselves regarding this service care model to successfully implement the use of telehealth in dysphagia management, particularly during challenging times such as the acute treatment interval during head and neck radiotherapy.

Lessons from this study offer useful information that may not only help improve delivery of care using telehealth in the PRO-ACTIVE trial but also inform best practices for dysphagia management using telehealth. Our work supports previous evidence that a telehealth approach was a beneficial alternate model to support patients during service constraints [16], particularly during the COVID-19 pandemic. Our findings complement previous study reports that most patients are comfortable receiving services via telehealth and appreciate the value of telerehabilitation [34]. Limitations related to technical factors and accessibility identified from the participants’ viewpoints have been reported previously [25] and helps to understand which patients might benefit more from an in-person session compared to a virtual therapy model. It is important for clinicians to evaluate patients’ individual challenges and clinical needs and adopt the use of telehealth only if it is judged to be in the best interests of the patient given their clinical scenario.

Despite a robust review of the participants’ interviews, there are some limitations to report for this qualitative study. This study included a very small sample of only eleven participants making it difficult to draw conclusions about the impact of a previous in-person visit(s) before the telehealth appointment. The authors did not have the opportunity to continue with interviews to confirm data saturation. This is a study limitation making it difficult to conclude that authors have indeed captured all possible key messages from a patient perspective regarding telehealth. The small sample size of this study also limited the authors from doing a comprehensive compare/contrast of the participant responses based on age, sex, and other factors such as socioeconomic status, ethnicity, residential geography and co-habitation status, all of which have the potential to impact participant responses. The authors did compare/contrast responses from Canada vs USA and found no differences although the dataset is too small to conclude that differences do not exist. Participants were included from a tertiary care academic institute in Toronto, ON Canada (PM/UHN) and an academic comprehensive cancer center facility in Houston, Texas, US (MMDACC). In future studies, with a larger sample, it would be interesting to see if patient perspectives differ between those enrolled at an academic versus a community facility. Participants were interviewed between 3 to 9 months following their last telehealth SLP therapy session and some interviews were more detailed than others. This large time gap between the telehealth SLP session and the interview has the potential risk of recall bias with less detail about the SLP sessions being described when the session occurred some time ago.

This study was primarily driven by the changes that occurred in response to the COVID-19 pandemic leading to a rapid shift in SLP care delivery. Perspectives of patients receiving telehealth rapidly implemented during a pandemic response may reflect more real-word, pragmatic perspective than prior controlled research in this area. However, it is also important to consider that despite the several benefits and advantages of telehealth highlighted in this study, there isn’t enough data to conclude that these participant views can be generalized beyond the context of the pandemic. Nonetheless, current literature presents promising data, indicating that telehealth interventions may be both effective and cost-efficient in the management of head and neck patients [35]. In fact, the COVID-19 pandemic resulted in widespread telehealth implementation in healthcare increasing access to care and reducing health disparities in under-represented minority and vulnerable patient populations [36]. In the research arena, the concept of a decentralized or hybrid clinical trial has been adopted including remote collection and assessment of data, that potentially reduce patient burden, increase patient enrollment and retention, and also improve efficiency of trial workflow efficiency [37]. Given the exponential growth in the use of technology in modern medicine and the extensive interest and research in the field of telehealth, the likelihood of using a telehealth approach for dysphagia management in future is high. Learning from the patient point of view what facilitates effective exchange between an SLP and the patient, and what barriers exist, can help ensure best practices in utilizing the telehealth approach in the PRO-ACTIVE trial as well as in future uptake and application of a successful intervention for dysphagia management in the head and neck cancer population.

## Supplementary Information

**Additional file 1.** PRO-ACTIVE Ancillary 01 – Telehealth Study Interview Script, Exploring patient experiences with a telehealth approach for the PRO-ACTIVE trial intervention in head and neck cancer patients.
