# Supplementary material for: Exploring patient experiences with a telehealth approach for the PRO-ACTIVE trial intervention in head and neck cancer patients
Source: BMC Health Serv Res. 2022 Sep 30;22:1218. doi: 10.1186/s12913-022-08554-6 (PMC9523628; doi:10.1186/s12913-022-08554-6)
Supplement: Supplementary file 1 — Additional file 1. PRO-ACTIVE Ancillary 01 – Telehealth Study Interview Script, Exploring patient experiences with a telehealth approach for the PRO-ACTIVE trial intervention in head and neck cancer patients. [file 12913_2022_8554_MOESM1_ESM.docx]

**Interview script – Experiences with PRO-ACTIVE intervention via telehealth**

Thank you very much for agreeing to talk with me about your experiences with the PRO-ACTIVE speech therapy session(s) using the X system, which going forward we will simply call ‘telehealth’. We are interested in hearing what it was like for you to use the telehealth or videoconference system, what you found worked well, and where improvements could be made.

There are no right or wrong answers to the questions. You can choose to not answer any of them if you do not want to answer. You can share as much or as little as you wish. We had thought the conversation would take about half an hour to an hour, depending on how much you want to share with me.

*Do you have any questions before we begin?*

I understand that you went through X sessions with the speech language pathologist using the telehealth approach. During those sessions there was both assessment of how you were getting along and some instruction about [eating/exercising].

*First of all, tell me about what it was like for you to use the telehealth approach?*

*When you first heard that the sessions would be via telehealth, what was your reaction? What did you think about that?*

*How much experience had you had with using telehealth prior to these sessions?*

*What did you expect the telehealth sessions would be like?*

*What preparation did you have to do before the telehealth sessions began?*

*What were the telehealth sessions actually like? What happened during the telehealth sessions?*

*What made it easy for you during the telehealth sessions? What made it difficult for you during the telehealth sessions?*

*How did you feel at the end of the first session? How did you feel at the end of the rest of the telehealth sessions? [Probe: How satisfied were you with the sessions?]*

*How clear was the information that was shared with you during the telehealth session? Were there any points that were unclear or confusing?*

*How confident were you about what you had learned during the telehealth session and whether you could do it on your own?*

*What do you think should be changed when delivering the therapy sessions using telehealth [Probe: the preparation? The delivery?]*

*When you think about the session you had with the speech language pathologist in person at the hospital, how did it compare to the session via telehealth? [Probe: Was it more effective or less effective?]*
